# Identification of *Schizosaccharomyces pombe* in the guts of healthy individuals and patients with colorectal cancer: preliminary evidence from a gut microbiome secretome study

**DOI:** 10.1186/s13099-018-0258-5

**Published:** 2018-07-10

**Authors:** Siok-Fong Chin, Putri Intan Hafizah Megat Mohd Azlan, Luqman Mazlan, Hui-min Neoh

**Affiliations:** 1 UKM Medical Molecular Biology Institute, Universiti Kebangsaan Malaysia, Cheras, Kuala Lumpur, Malaysia; 2 Department of Surgery, Faculty of Medicine, Universiti Kebangsaan Malaysia Medical Centre, Cheras, Kuala Lumpur, Malaysia

**Keywords:** *Schizosaccharomyces pombe*, Human gut, Microbiome, Secretome, Colorectal cancer

## Abstract

Over the years, genetic profiling of the gut microbiome of patients with colorectal cancer (CRC) using genome sequencing has suggested over-representation of several bacterial taxa. However, little is known about the protein or metabolite secretions from the microbiota that could lead to CRC pathology. Proteomic studies on the role of microbial secretome in CRC are relatively rare. Here, we report the identification of proteins from *Schizosaccharomyces pombe* found in the stool samples of both healthy individuals and patients with CRC. We found that distinctive sets of *S. pombe* proteins were present exclusively and in high intensities in each group. Our finding may trigger a new interest in the role of gut mycobiota in carcinogenesis.

## Gut microbiome and colorectal cancer

Dysbiosis of the gut microbiome has been postulated to be the causative event in colorectal cancer (CRC). For many years, most microbiome studies focused primarily on genetic sequencing and the subsequent identification of gut bacteria; however, the role of bacteria in CRC carcinogenesis remains unclear. We hypothesised that protein secretions from these microorganisms play a role in the pathophysiology of CRC. We aimed to identify and compare the secreted proteins released from the human gut microflora by assessing the stool samples of both patients with CRC and healthy control individuals.

## Faecal sample extraction and protein identification

A pilot study was performed at the endoscopy unit and surgery and medical clinics at the UKM Medical Centre, Kuala Lumpur, from 2016 to 2017. Stool samples from 26 patients with clinically diagnosed CRC and 20 non-CRC control individuals were collected from the UKM Medical Centre. The samples were homogenised and filtered, followed by precipitation with acetone, reduction with dl-Dithiothreitol, alkylation with iodoacetamide and digestion with trypsin. The resulting peptides were analysed by reverse-phase LC–ESI–MS/MS using Eksigent NanoLC system connected to a quadrupole time-of-flight 5600+ TripleTOF™ mass spectrometer (AB Sciex, USA). Briefly, mass spectra were acquired in the data-dependent mode (250 ms accumulation time per spectrum and mass range of 300–1250 *m/z*) to obtain MS/MS spectra for the most abundant parent ions following each survey MS1 scan. For each mass spectrum, a maximum of 50 precursors with a charge state between 2 and 4 were selected for fragmentation. The tandem mass spectrum was acquired in the high-sensitivity mode, in which the signals were accumulated for a minimum of 50 ms per spectrum, and the dynamic exclusion time was set at 24 s.

The mass spectrum datasets were searched using MaxQuant against the microbial UniProt Fasta database (accessed on 18 September 2017). Statistical analyses were performed using the Statistical Package for Social Sciences (SPSS) version 22. Functional and integrative analyses of the identified proteins were performed using the Database for Annotation, Visualisation and Integrated Discovery (DAVID) [1] and MetaboAnalyst 4.0 [2] online tools, respectively.

## Secretome of gut microbiome in healthy individuals and patients with CRC

We identified 2132 proteins secreted by the gut flora (1370 from bacteria; 589 from fungi; 112 from archaea; 45 from viruses to 16 from parasites), with 96 proteins specific to CRC. In contrast, 2035 proteins were exclusive to the non-CRC controls. Only one bacterial protein from *Borrelia recurrentis* was commonly secreted in both CRC and non-CRC groups. Interestingly, we found that vast protein secretions were mapped to *S. pombe*, which were not previously reported in the human gut. *S. pombe*, a fission yeast, is traditionally used in brewing. Its 50 genes are similar to human genes associated with diseases, with 23 genes similar to human cancer genes [3]. This yeast has been widely used as a model organism to study human diseases and pathological events, such as cancer and ageing [4, 5]. To the best of our knowledge, no previous study has shown the association between *S. pombe* and human diseases.

**Fig. 1:**
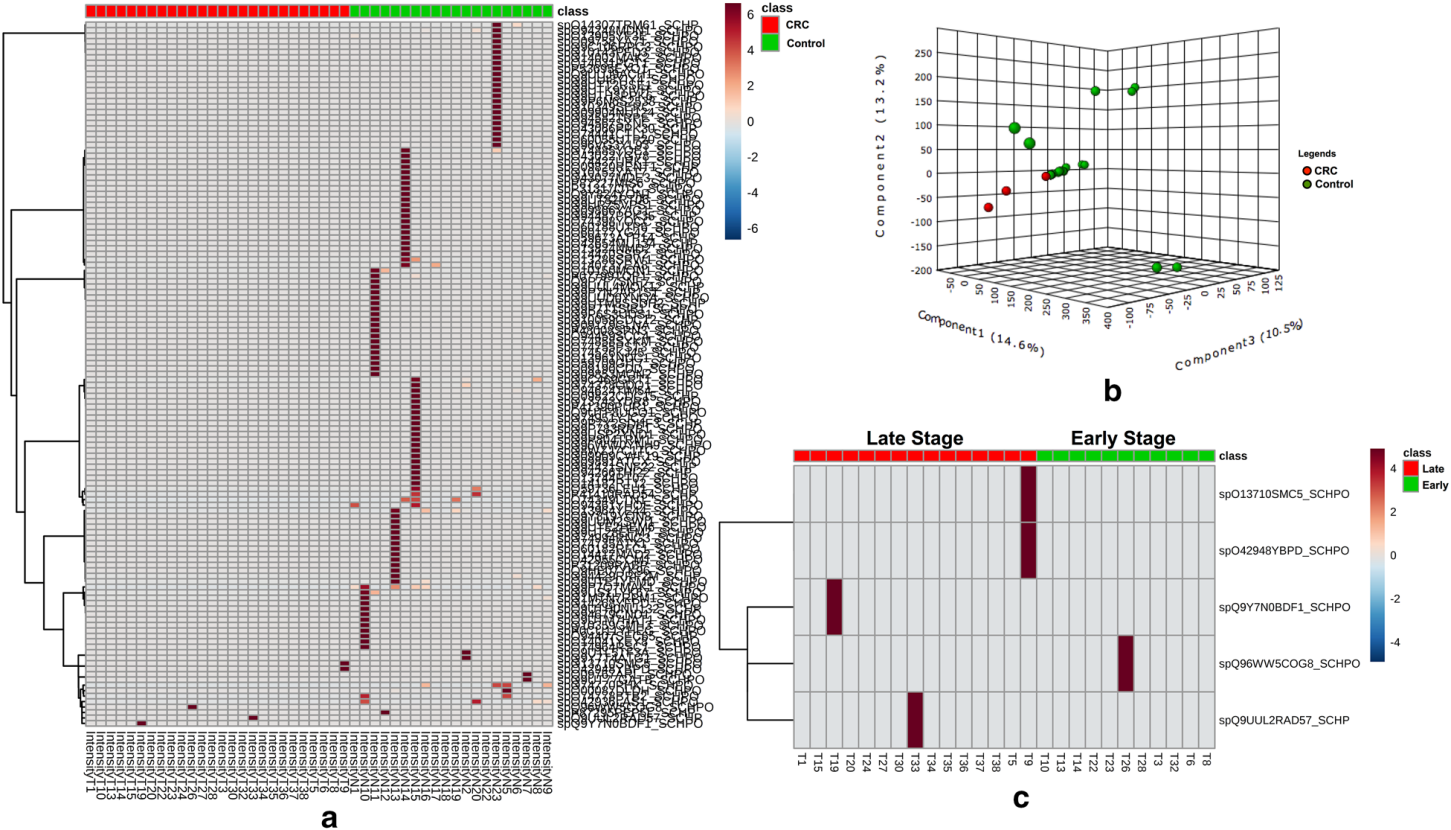
Distribution of secretome proteins identified from *S. pombe* in the gut of patients with CRC and control individuals. **a** Heat-map analysis revealed individual variation in secreted proteins by *S. pombe*; **b** discriminant analysis showed that proteins from *S. pombe* were well-discriminated between the CRC and control groups; **c** different sets of proteins were identified from *S. pombe* during the late and early stages of CRC

In our study, a total of 124 proteins secreted by *S. pombe* were exclusively found in the control samples. Five proteins found in the CRC samples were successfully mapped to the yeast. The distribution of the secretome proteins identified from the yeast was skewed towards the control samples compared with the CRC samples (Fig. 1). We also observed great variation in the secretome of *S. pombe* in the studied individuals, regardless of the presence or absence of CRC or early- or late-stage CRC, as revealed by discriminant and heat-map analyses. However, all the secretome proteins from *S. pombe* identified in the CRC were of high intensity, with four proteins exclusively secreted in the late stage of CRC (DNA repair protein rhp57; SWR1 complex bromodomain subunit bdf1; structural maintenance of chromosomes protein 5 and uncharacterised WD repeat-containing protein C16H5.13). In contrast, the conserved oligomeric Golgi complex subunit 8 was secreted in the early stage of CRC.

## Conclusion

The importance of mycobiota in gut health has been recently postulated. The study of these fungi may provide new therapeutic approaches. Of note, the current probiotics paradigm has shifted from the use of bacteria to yeast [6]. However, little is known about the role of these naturally occurring mycobiota in the gut microenvironment and their effects on the host (e.g., contributing to health or diseases), particularly chronic effects at the molecular level. We suggest that distinctive sets of *S. pombe* proteins be used as markers to distinguish between individuals with and without CRC. It is still unknown if the proteins secreted by *S. pombe* identified in this study result in tumorigenesis or vice versa. Thus, further investigation is warranted.

## Abbreviations

CRC: colorectal cancer
*S. pombe*: *Schizosaccharomyces pombe*
SPSS: Statistical Package for the Social Sciences
DAVID: Database for Annotation, Visualisation and Integrated Discovery
